# Supplementary material for: Demonstration of valley anisotropy utilized to enhance the thermoelectric power factor
Source: Nat Commun. 2021 Sep 17;12:5408. doi: 10.1038/s41467-021-25722-0 (PMC8448840; doi:10.1038/s41467-021-25722-0)
Supplement: Supplementary file 1 — Supplementary Information [file 41467_2021_25722_MOESM1_ESM.pdf]

Demonstration of Valley Anisotropy Utilized to Enhance the Thermoelectric Power Factor

Airan Li[1], Chaoliang Hu[1], Bin He[2], Mengyu Yao[2], Chenguang Fu[1*], Yuechu Wang[1], Xinbing Zhao[1], Claudia Felser[2] & Tiejun Zhu[1*]

[1]State Key Laboratory of Silicon Materials, School of Materials Science and Engineering, Zhejiang University, 310027 Hangzhou, China. [2]Max Planck Institute for Chemical Physics of Solids, Nöthnitzer Str. 40, 01187 Dresden, Germany. These authors contributed equally: Airan Li, Chaoliang Hu. Correspondence and requests for materials should be addressed to C. F. (email: chenguang_fu@zju.edu.cn) or to T. Z. (email: zhutj@zju.edu.cn)

Supplementary Note 1

Single parabolic band model. The single parabolic band model is a widely used to understand the charge transport properties in thermoelectric materials. Under the assumption of the acoustic phonon scattering dominated carrier transport, *S* can be expressed as:

$$S = \frac{k}{e}\left(\frac{2F_1}{F_0} - \eta\right) \quad (1)$$

where *k* is Boltzmann constant, *e* is elemental charge, η is reduced Fermi level, F_j is Fermi integrals:

$$F_j(\eta) = \int_0^{\infty} \frac{\xi^j d\xi}{1+exp(\xi-\eta)} \quad (2)$$

ξ is the reduced energy level, The η can be obtained by solving the above equations with the measured *S*. And then, the effective mass of density of states (DOS) m_d^* can be estimated using the chemical carrier density *n* by the following formula:

$$n = 4\pi\left(\frac{2md^*kT}{h^2}\right)^{3/2} F_{1/2} \quad (3)$$

where *T* is absolute temperature, *h* is Planck constant.

The measured Hall carrier density n_H is related to the chemical carrier density *n* via $n_H = n/r_H$, where the Hall factor r_H is given by:

$$r_H = \frac{3}{2} F_{1/2} \frac{F_{-1/2}}{2F_0^{\,2}} \quad (4)$$

After obtaining m_d^*, the n_H dependence of *S* can be plotted under a given *T* and the obtained m_d^*, namely, the so-called Pisarenko plot.

Meanwhile, the Lorenz number *L* can be calculated by:

$$L = \frac{k^2}{e^2} \frac{3F_0F_2 - 4{F_1}^2}{{F_0}^2} \tag{5}$$

Therefore, the lattice thermal conductivity κ_L can be obtained by subtracting the electronic contribution κ_e from the measured total thermal conductivity:

$$\kappa_L = \kappa - \kappa_e = \kappa - L\sigma T \tag{6}$$

Supplementary Figures and Tables

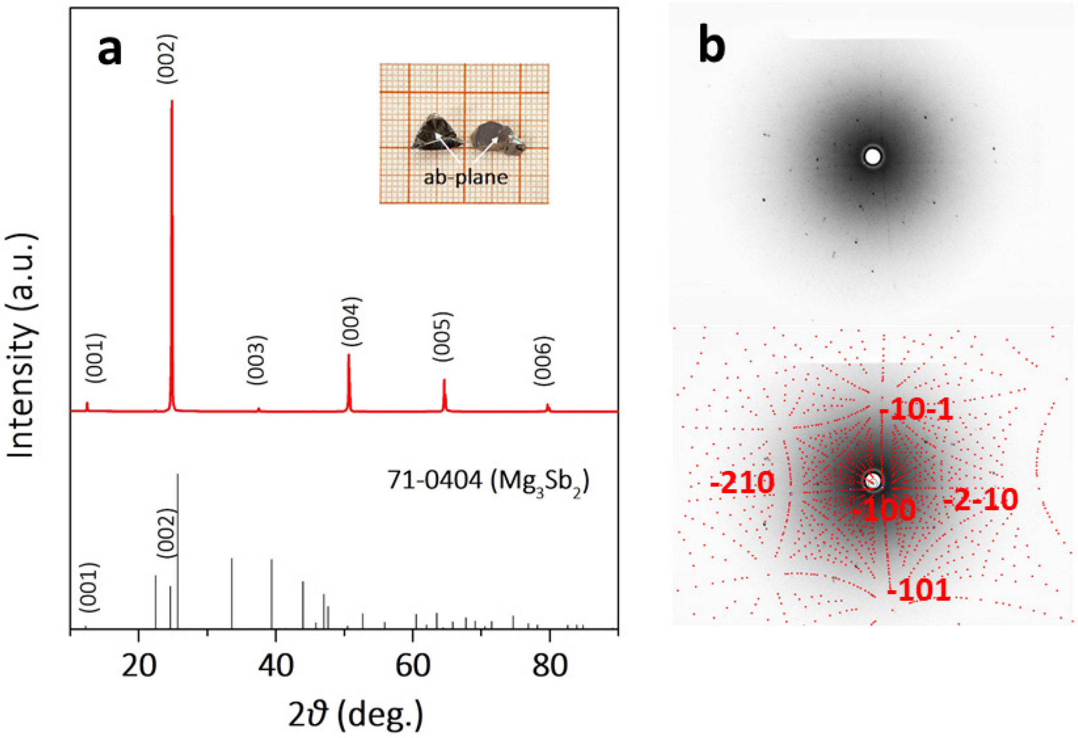

Supplementary Fig. 1 a The XRD pattern of the as-grown single crystal of Mg_3Sb_2 with a comparion with the simulated powder XRD pattern. **b** Laue diffraction of the as-grown crystal. The inset in **a** is the optical image of the single crystal. The cleavage surface is indicated by the white arrow in inset of **a**.

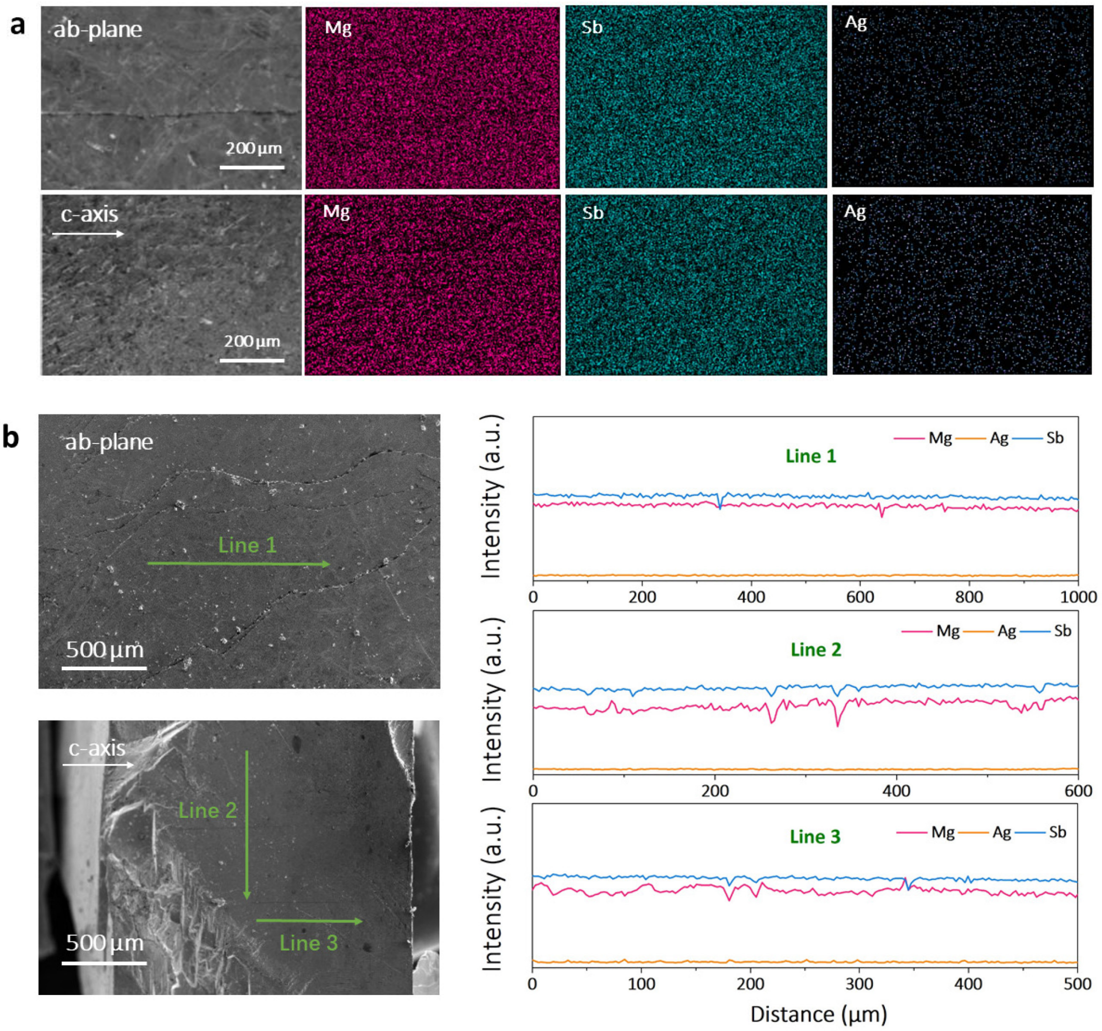

Supplementary Fig. 2 a The EDS mapping and **b** the line scanning along ab-plane and c-axis for the Mg_3Sb_2 single crystal.

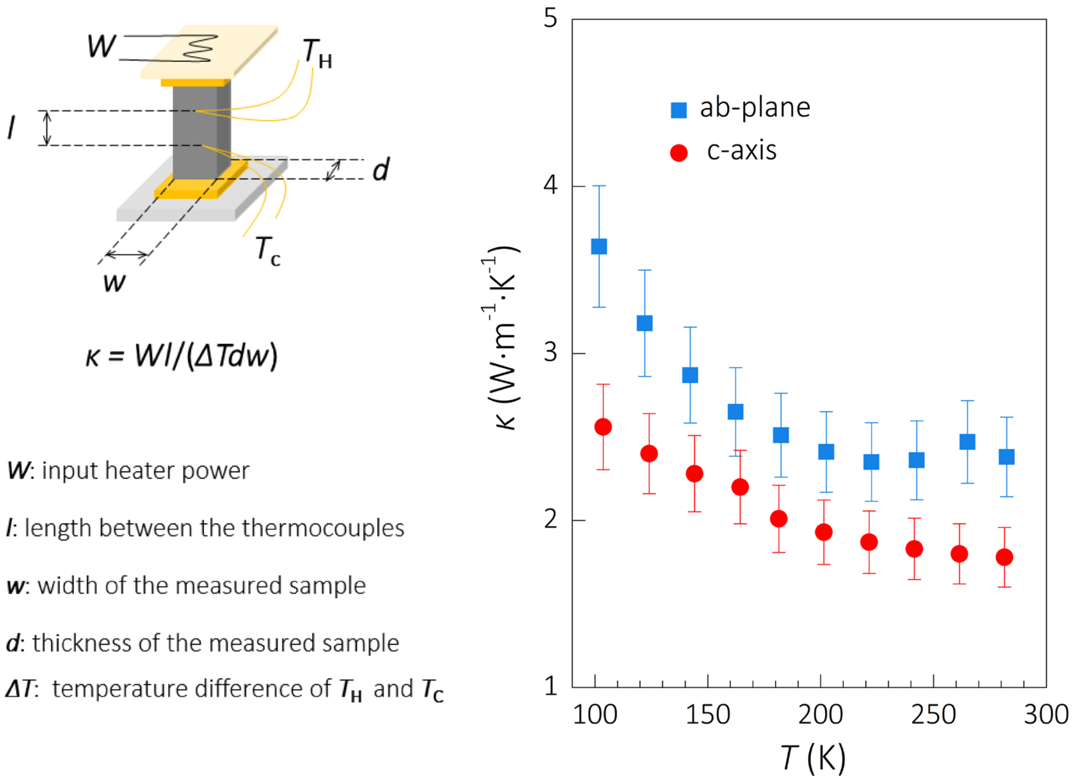

Supplementary Fig. 3 The schematic image of the measurement of the thermal conductivity for the studied single crystal along the ab-plane and c-axis.

Textured polycrystalline samples

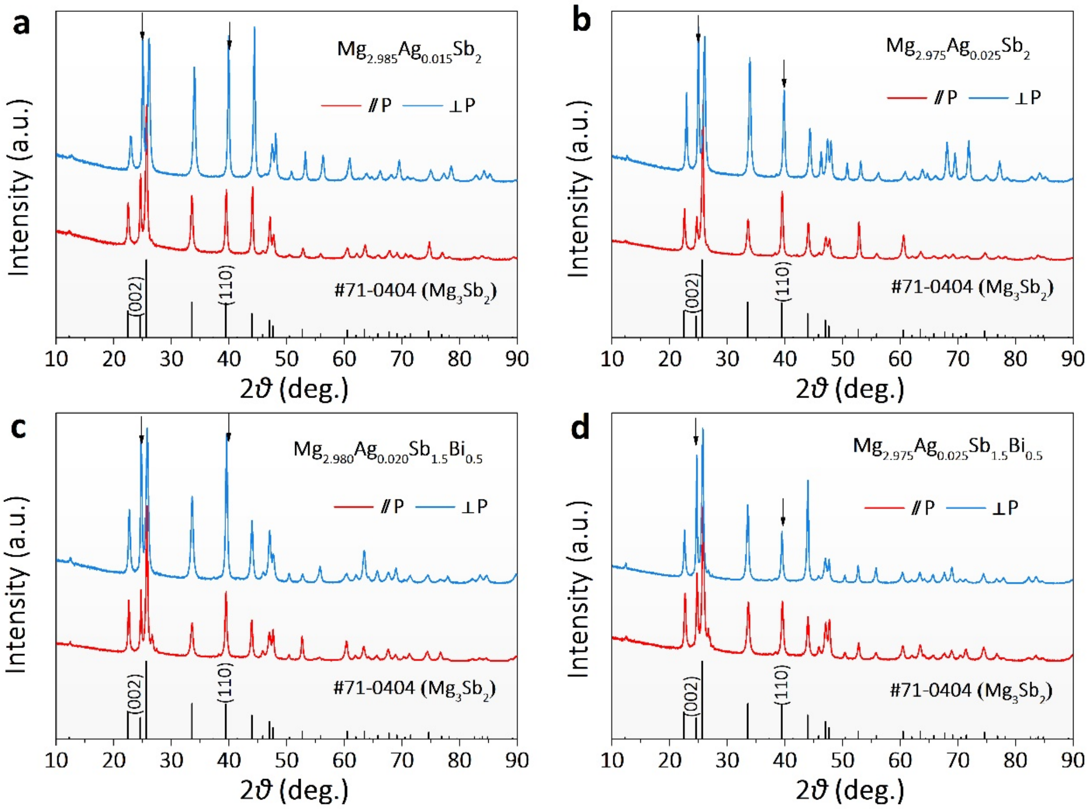

Supplementary Fig. 4 XRD patterns of the pressed ingots for **a** $Mg_{2.985}Ag_{0.015}Sb_2$, **b** $Mg_{2.975}Ag_{0.025}Sb_2$, **c** $Mg_{2.980}Ag_{0.020}Sb_{1.5}Bi_{0.5}$, **d** $Mg_{2.985}Ag_{0.015}Sb_{1.5}Bi_{0.5}$. All the samples display a textured feature.

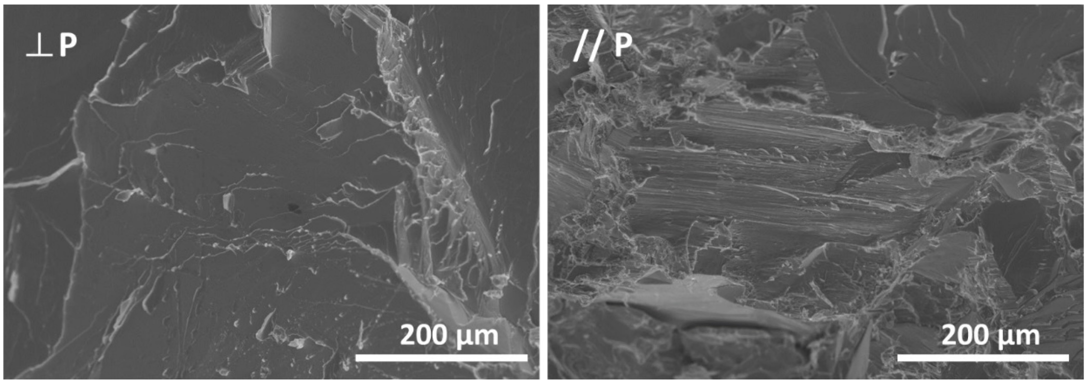

Supplementary Fig. 5 Secondary electron SEM images of the textured Mg_3Sb_2 samples.

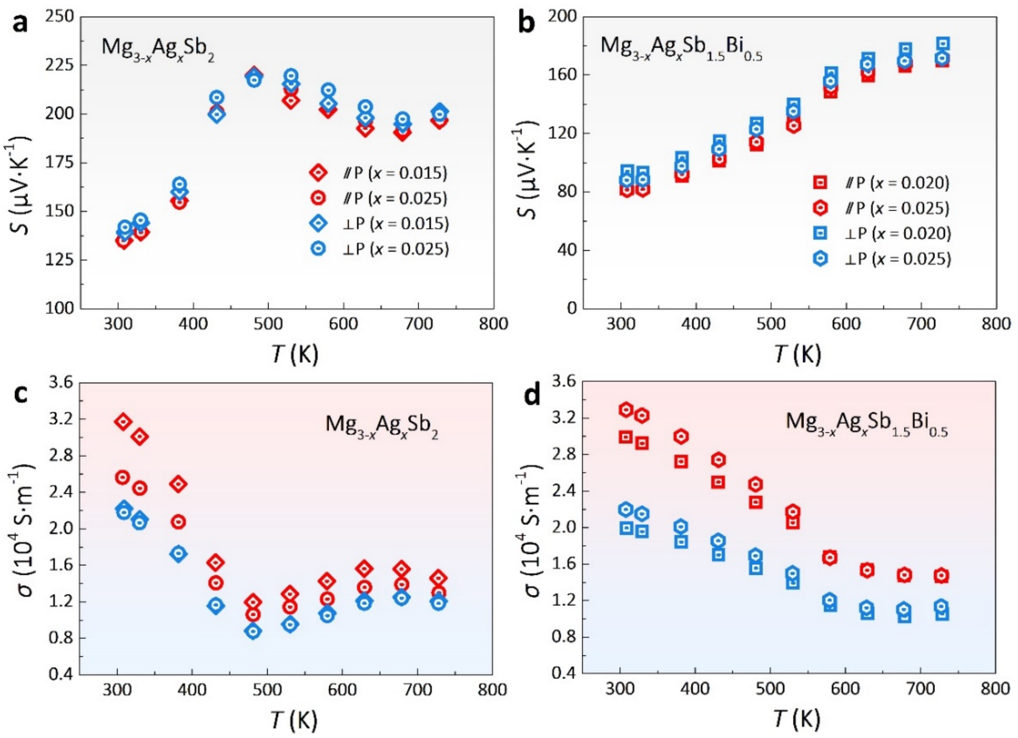

Supplementary Fig. 6 Temperature dependence of S and σ of the textured polycrystalline samples.

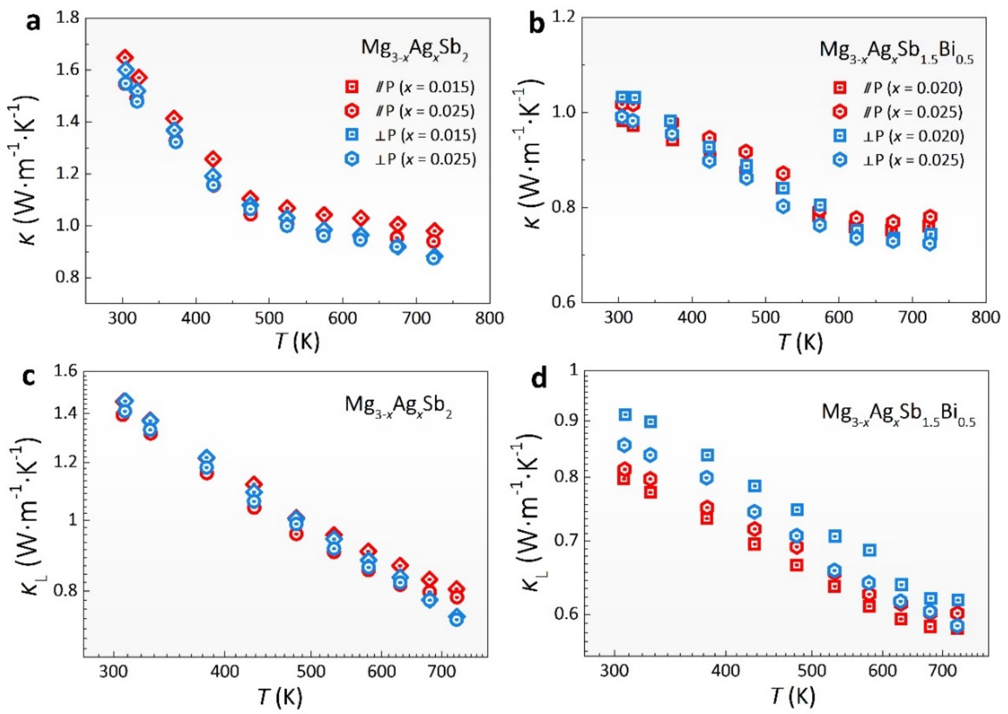

Supplementary Fig. 7 Temperature dependence of κ and κ_L of the textured samples with different contents of Ag and Bi.

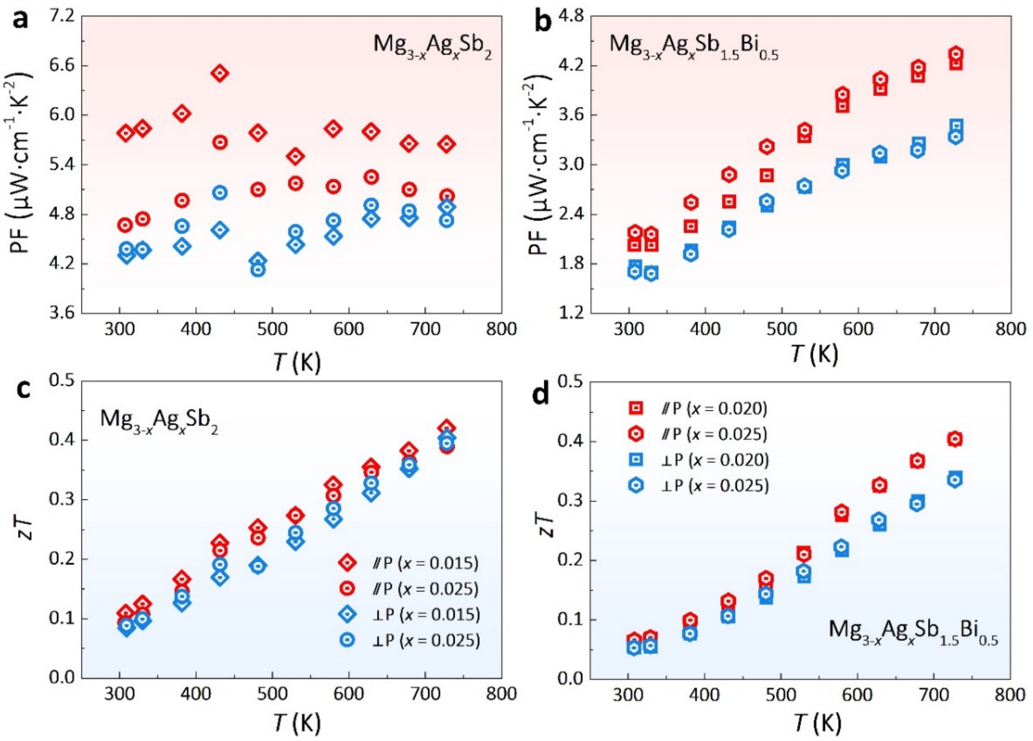

Supplementary Fig. 8 Temperature dependence of PF and zT of the textured samples with different contents of Ag and Bi.

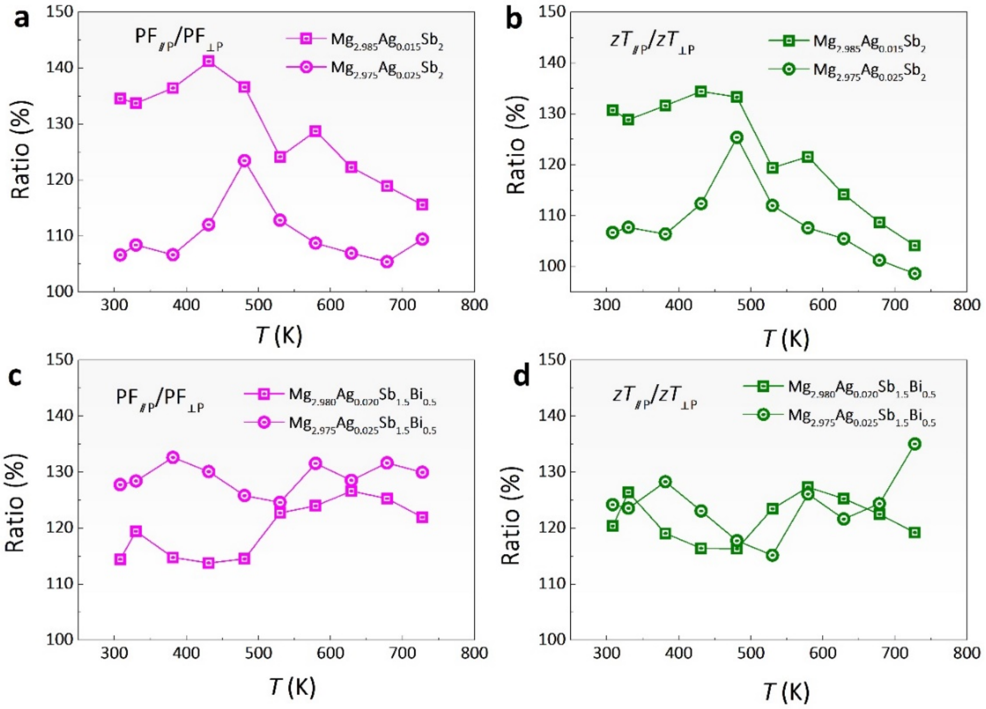

Supplementary Fig. 9 Temperature dependence of PF and *zT* ratio of textured polycrystalline samples with different contents of Ag and Bi.

Band structures of AB_2X_2 Zintl phase compounds with crystal splitting energy Δ > 0 eV.

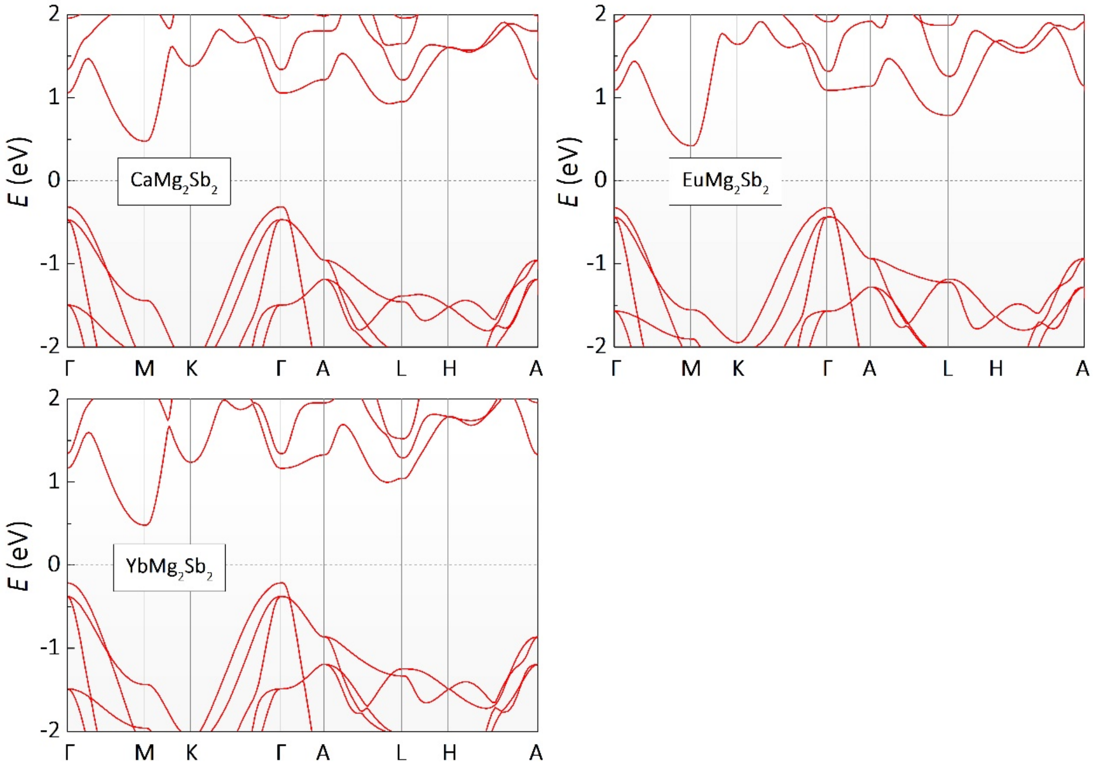

Supplementary Fig. 10 The band structure of $A Mg_2Sb_2$ (A = Ca, Eu, Yb).

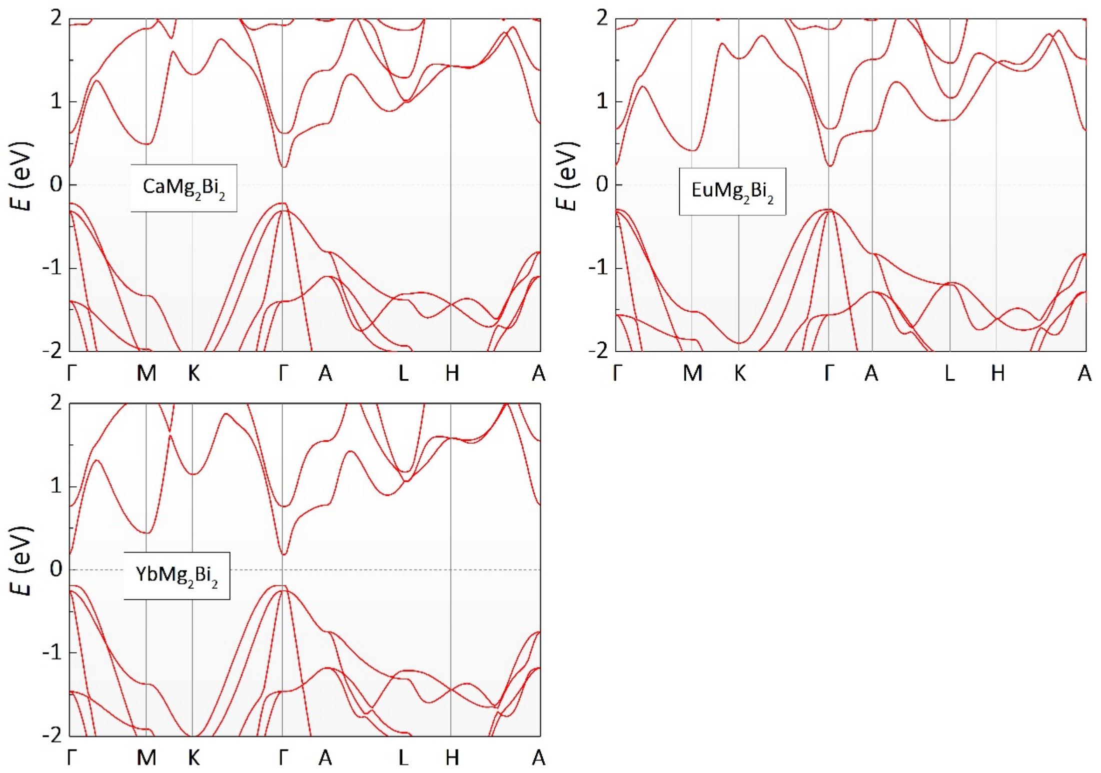

Supplementary Fig. 11 The band structure of AMg_2Bi_2 (A = Ca, Eu, Yb).

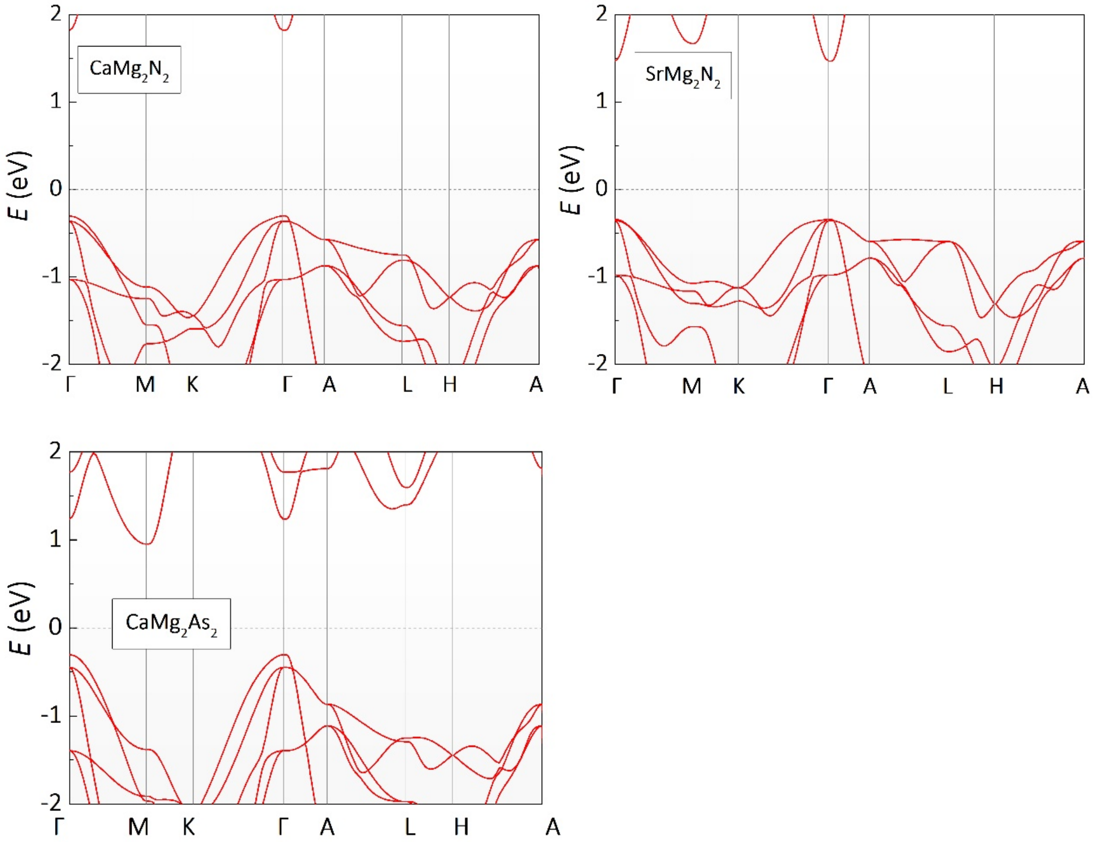

Supplementary Fig. 12 The band structure of $CaMg_2N_2$, $SrMg_2N_2$, $CaMg_2As_2$.

Band structures of AB_2X_2 Zintl phase compounds with Δ < 0 eV

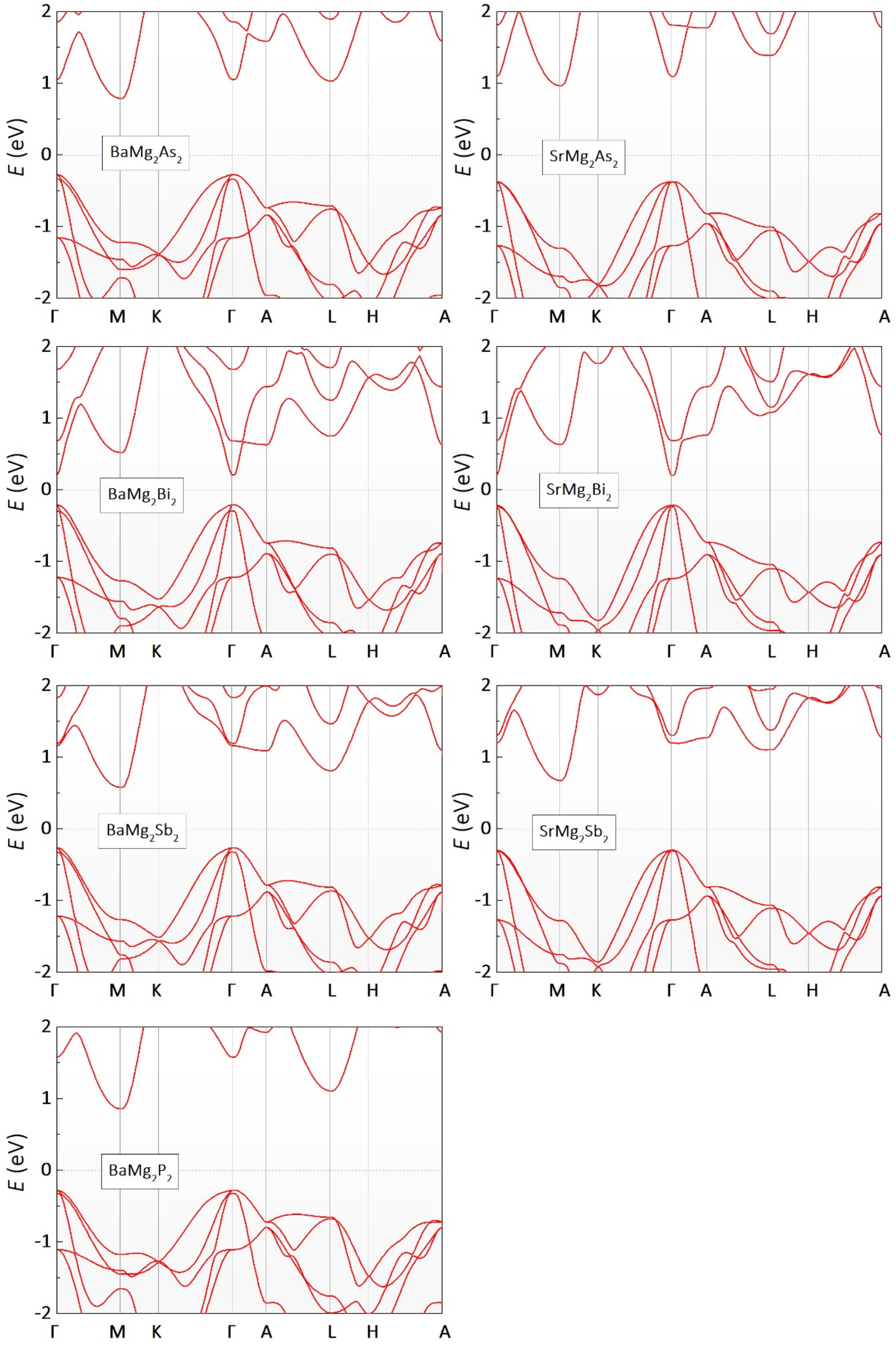

Supplementary Fig. 13 The band structure of $SrMg_2X_2$, $BaMg_2X_2$ (X = As, Bi, Sb); $BaMg_2P_2$.

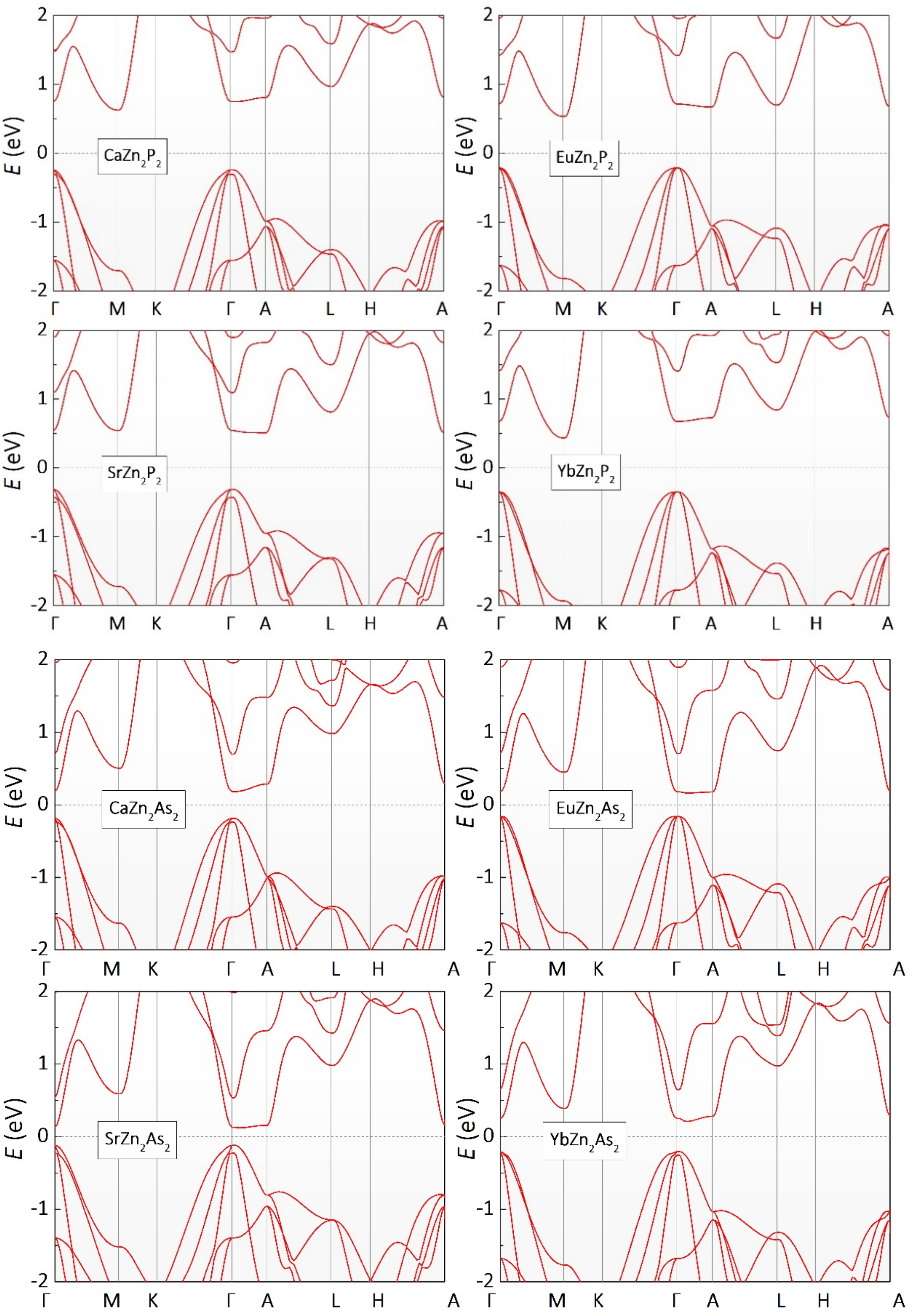

Supplementary Fig. 14 The band structure of AZn_2P_2, AZn_2As_2 (A = Ca, Eu, Sr, Yb).

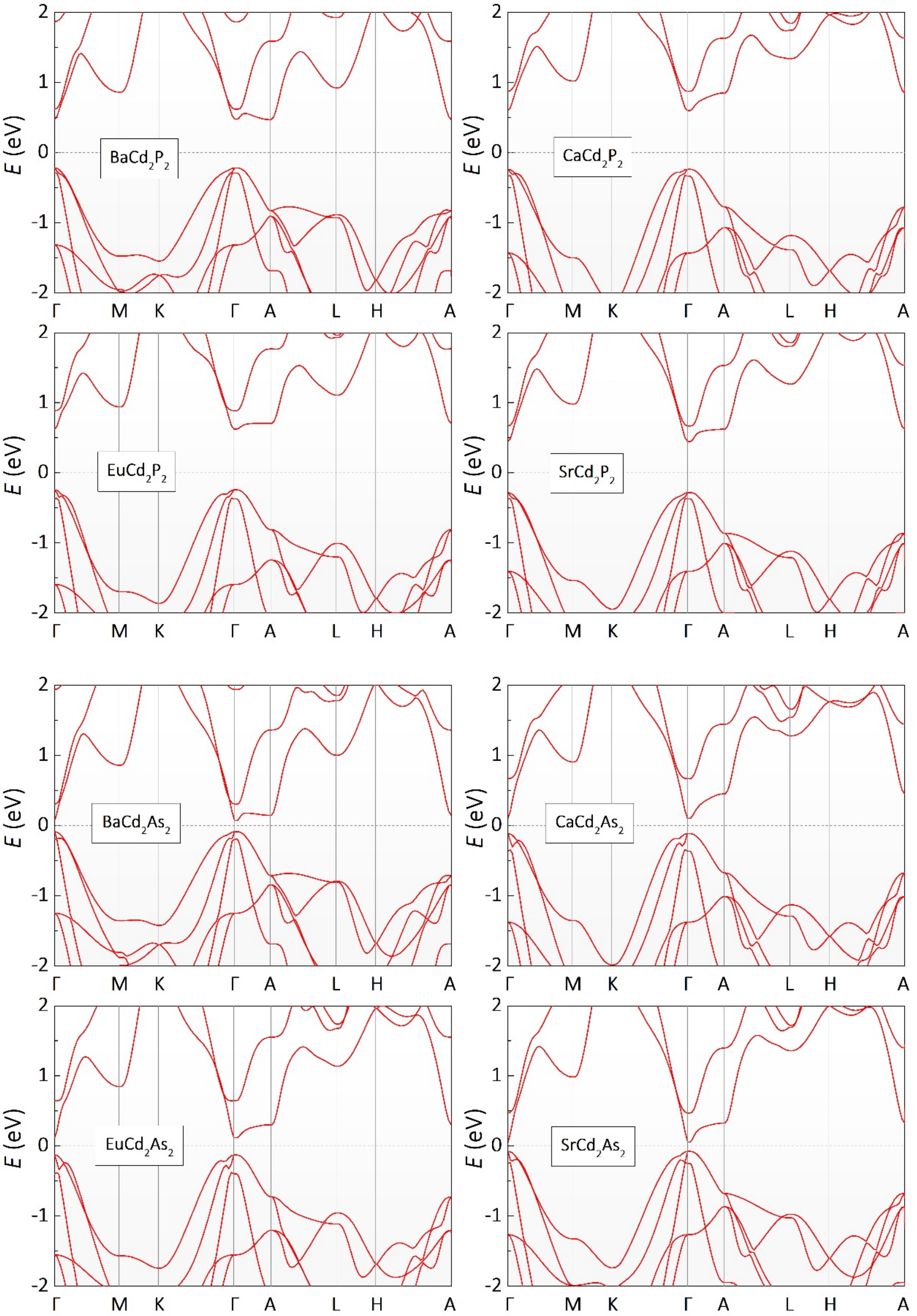

Supplementary Fig. 15 The band structure of ACd_2P_2, ACd_2As_2 (A = Ca, Ba, Eu, Sr).

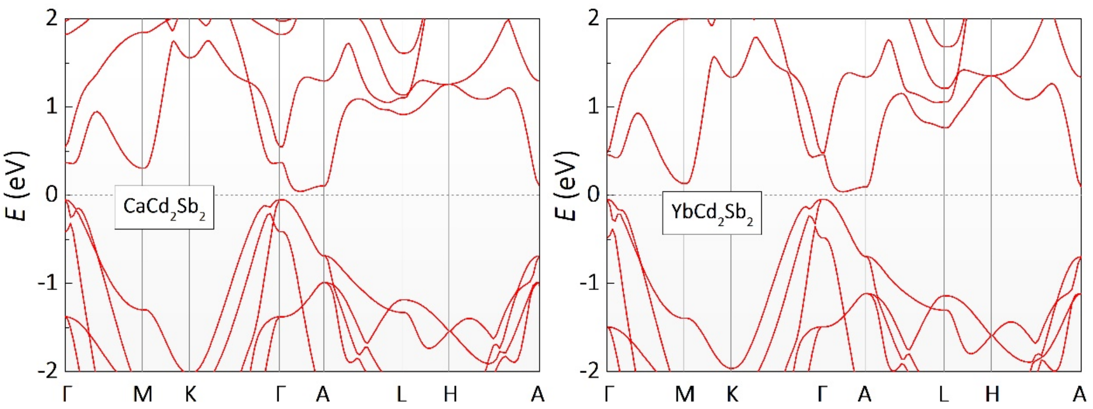

Supplementary Fig. 16 The band structure of $CaCd_2Sb_2$, $YbCd_2Sb_2$.

Band structures of *AB_2X_2* Zintl phase compounds without band gap.

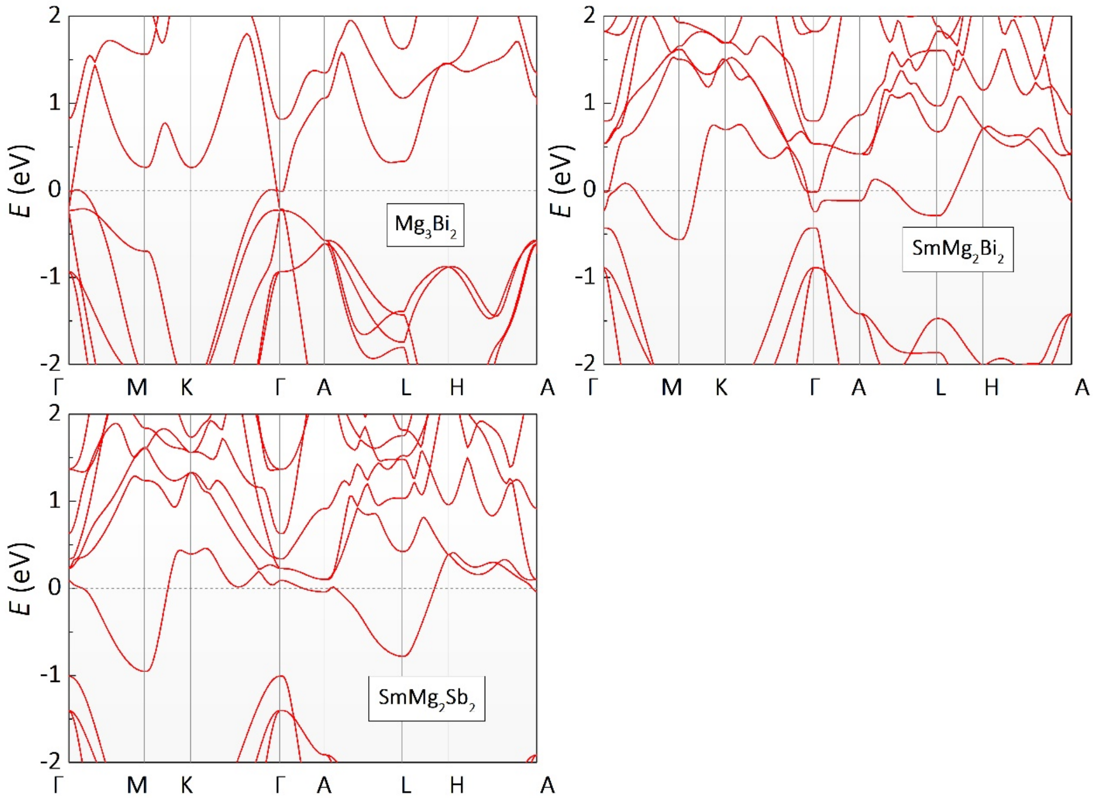

Supplementary Fig. 17 The band structure of Mg_3Bi_2, $SmMg_2Bi_2$, $SmMg_2Sb_2$.

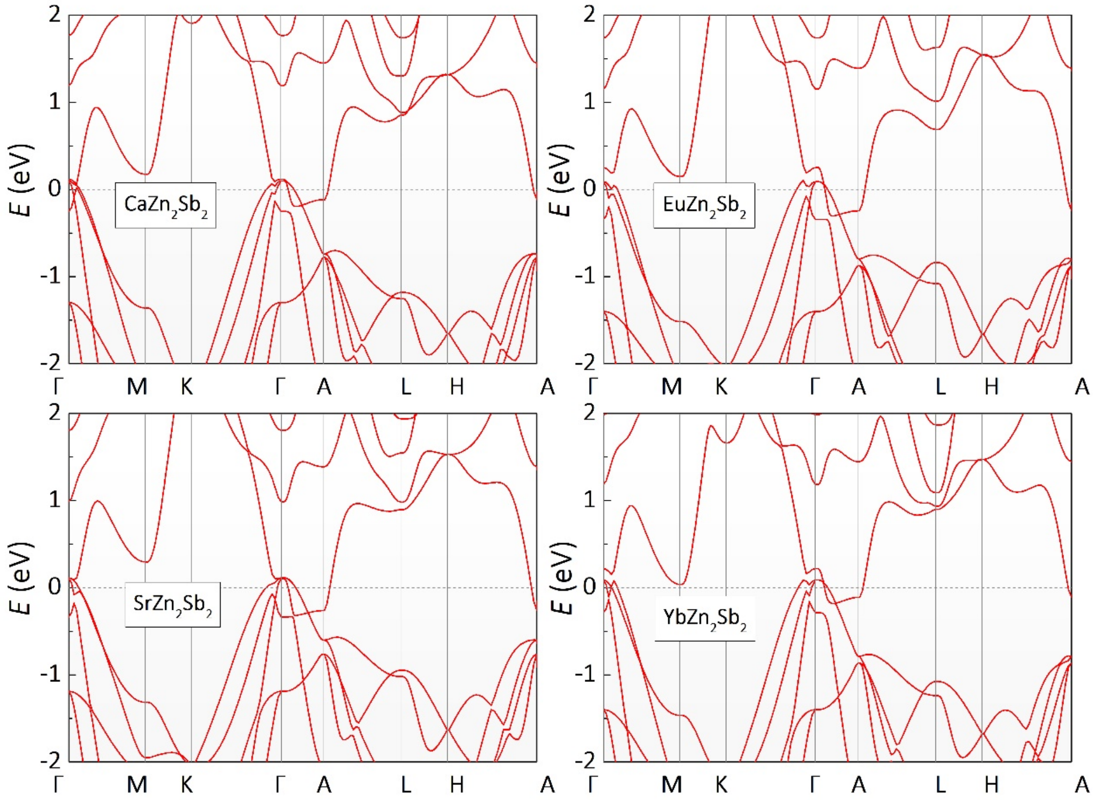

Supplementary Fig. 18 The band structure of AZn_2Sb_2(A = Ca, Eu, Sr, Yb).

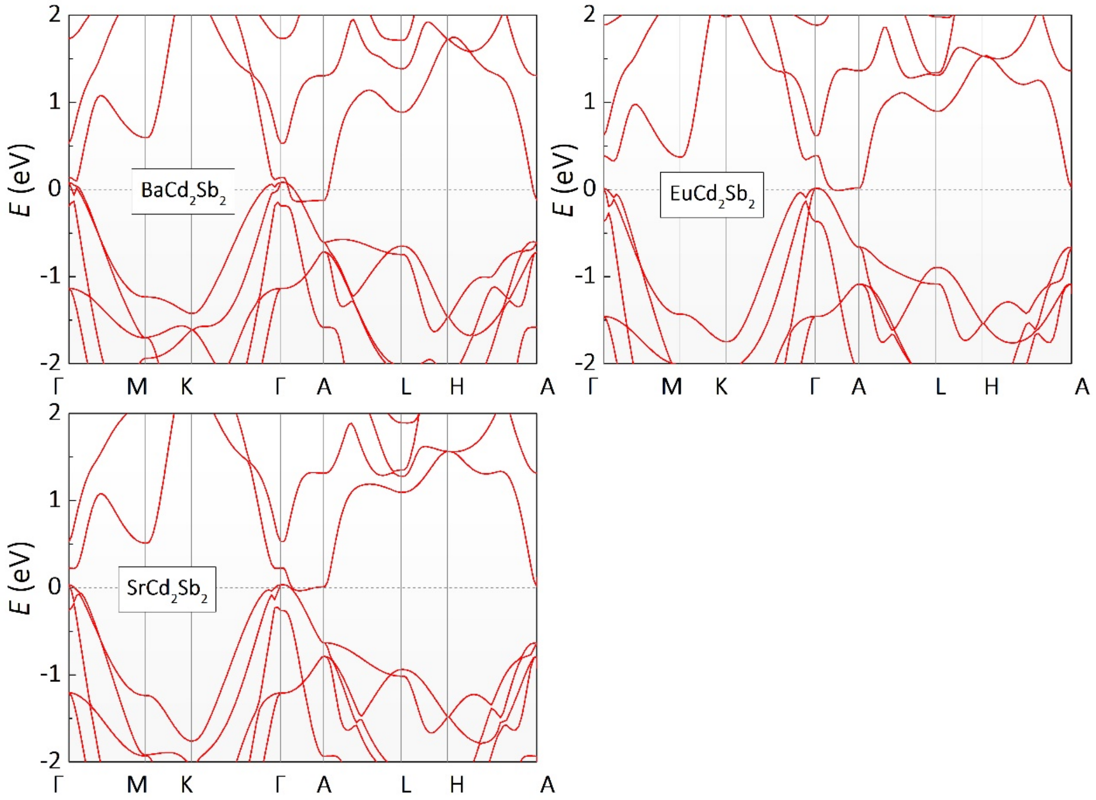

Supplementary Fig. 19 The band structure of ACd$_2$Sb$_2$(A = Ba, Eu, Sr).

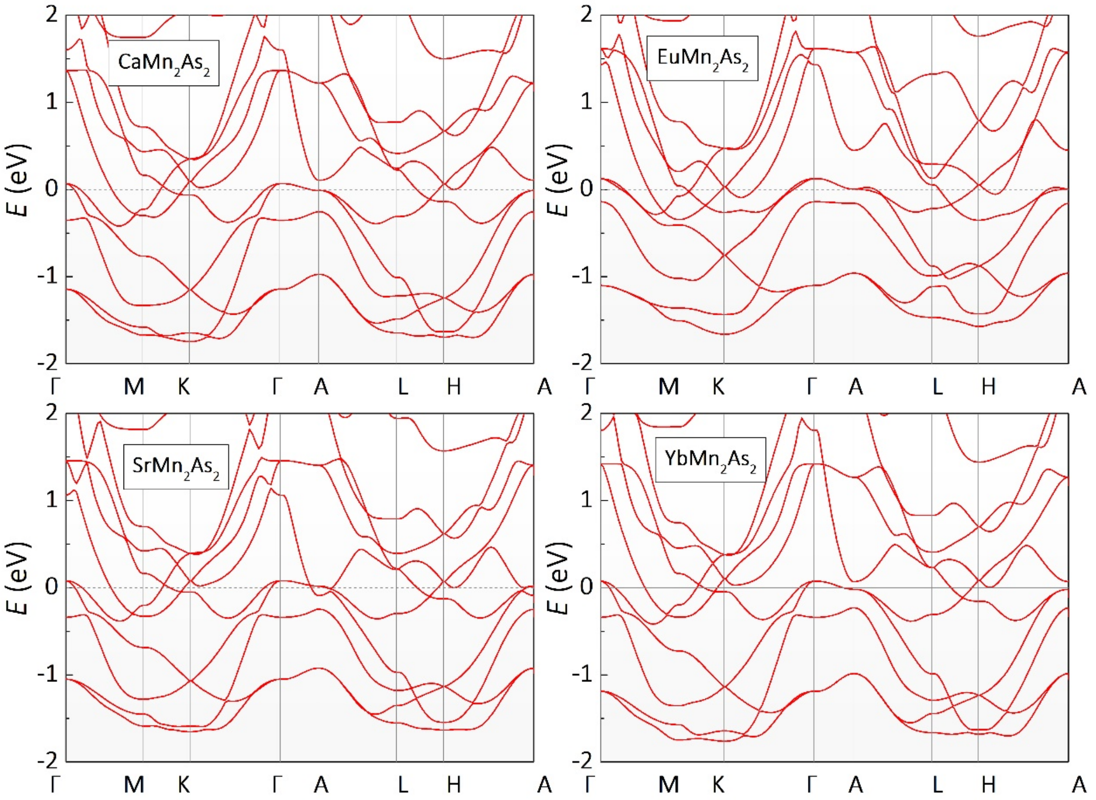

Supplementary Fig. 20 The band structure of $A\mathrm{Mn_2As_2}$(A = Ca, Eu, Sr, Yb).

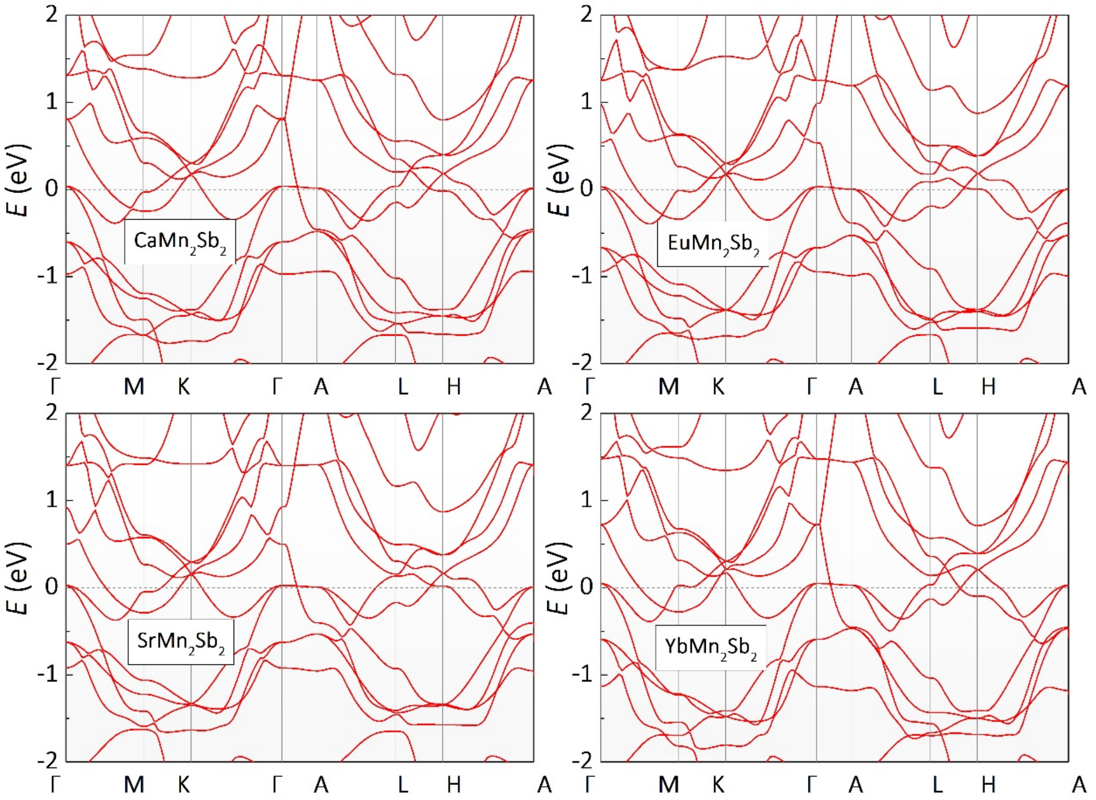

Supplementary Fig. 21 The band structure of $A\mathrm{Mn_2Sb_2}$(A = Ca, Eu, Sr, Yb).

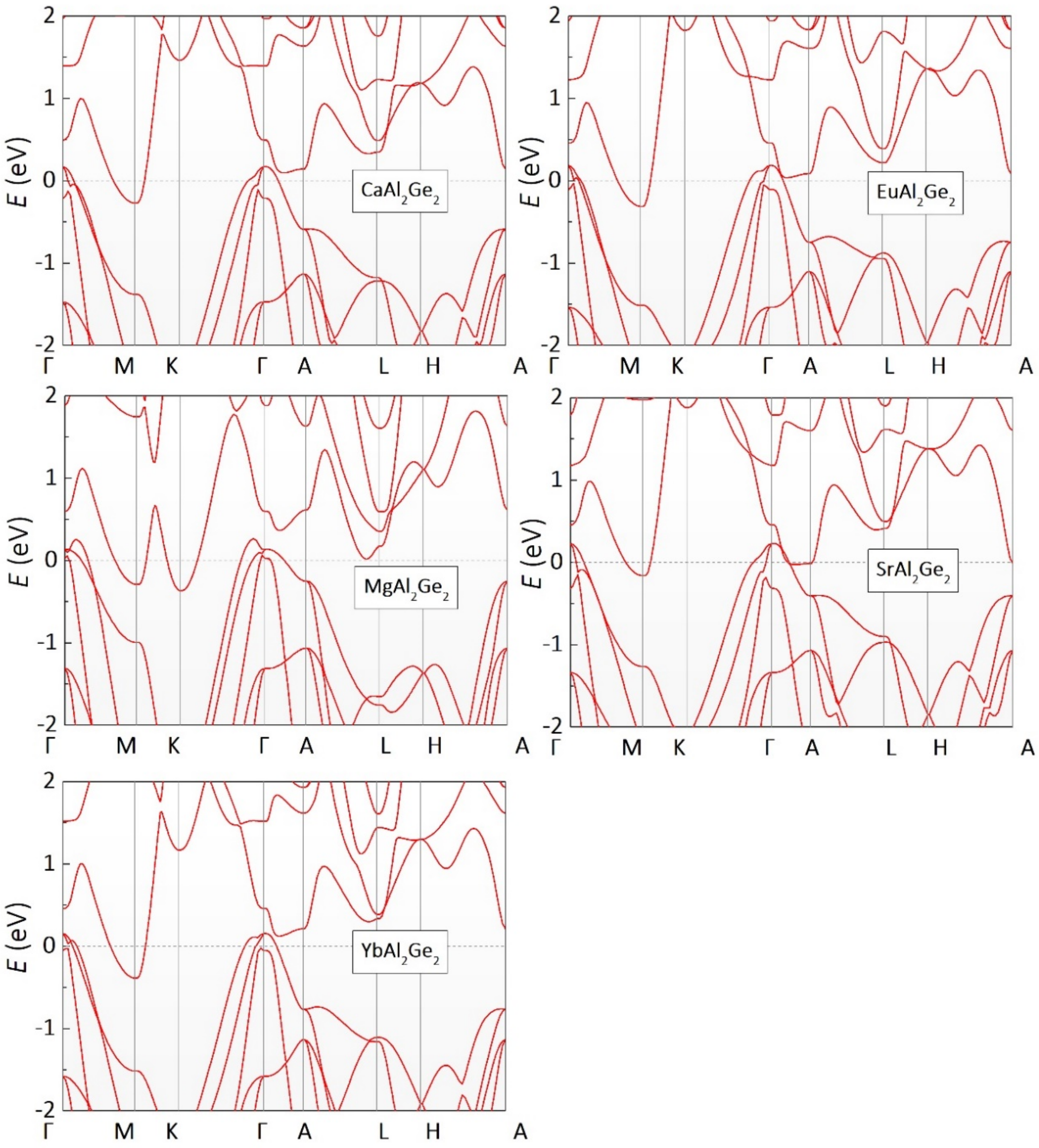

Supplementary Fig. 22 The band structure of AAl$_2$Ge$_2$ (A = Ca, Eu, Mg, Sr, Yb).

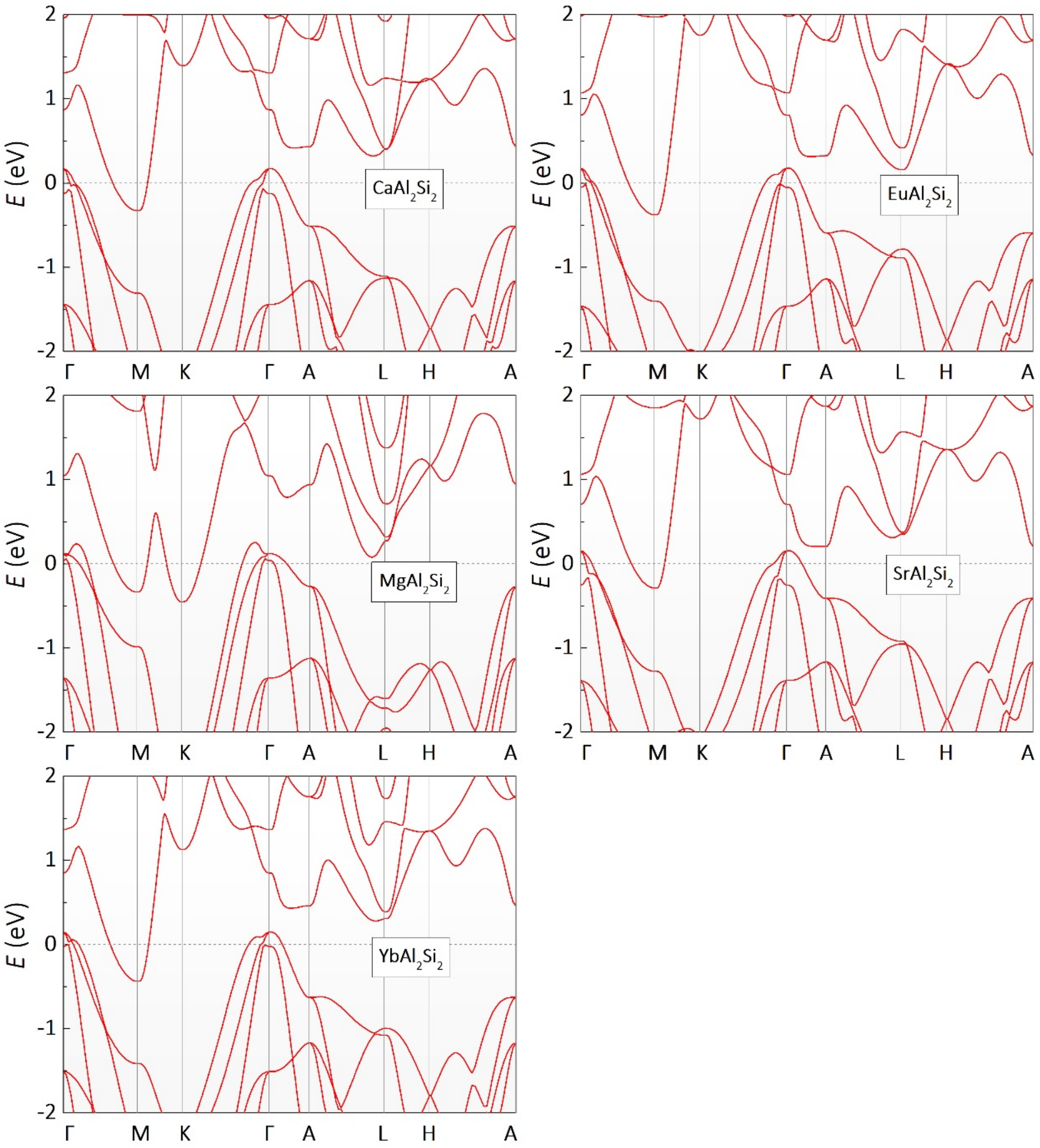

Supplementary Fig. 23 The band structure of AAl2Si2 (A = Ca, Eu, Mg, Sr, Yb).

Supplementary Table 1 Lattice parameters before and after relaxed for the studied AB_2X_2 Zintl phase compounds. The original lattice parameters are obtained from the Crystallography Open Database (http://www.nanocrystallography.org)

	Original		Relaxed (PBE)	
	a/Å	***c***/Å	***a***/Å	***c***/Å
$BaCd_2As_2$	4.513	7.674	4.589	7.755
$BaCd_2P_2$	4.402	7.557	4.452	7.609
$BaCd_2Sb_2$	4.770	8.080	4.861	8.132
$BaMg_2As_2$	4.480	7.740	4.517	7.788
$BaMg_2Bi_2$	4.860	8.220	4.919	8.334
$BaMg_2P_2$	4.367	7.580	4.389	7.614
$BaMg_2Sb_2$	4.770	8.100	4.822	8.207
$CaCd_2As_2$	4.391	7.184	4.445	7.215
$CaCd_2P_2$	4.277	7.031	4.311	7.044
$CaCd_2Sb_2$	4.649	7.597	4.716	7.618
$CaMg_2As_2$	4.340	7.130	4.378	7.144
$CaMg_2Bi_2$	4.730	7.680	4.786	7.724
$CaMg_2N_2$	3.540	6.091	3.535	6.069
$CaMg_2Sb_2$	4.660	7.580	4.691	7.579
$CaMn_2As_2$	4.230	7.030	3.848	7.087
$CaMn_2Bi_2$	4.630	7.640	3.858	8.940
$CaMn_2P_2$	4.096	6.848	3.756	6.700
$CaMn_2Sb_2$	4.540	7.480	3.900	8.252
$CaZn_2As_2$	4.162	7.010	4.192	7.022
$CaZn_2P_2$	4.038	6.836	4.043	6.834
$CaZn_2Sb_2$	4.441	7.464	4.491	7.469
$EuCd_2As_2$	4.439	7.328	4.474	7.247
$EuCd_2P_2$	4.325	7.183	4.338	7.085
$EuCd_2Sb_2$	4.699	7.725	4.750	7.645
$EuMg_2Bi_2$	4.772	7.837	4.799	7.792
$EuMg_2Sb_2$	4.695	7.724	4.717	7.694
$EuMn_2As_2$	4.287	7.225	3.907	7.114
$EuMn_2P_2$	4.143	7.034	3.763	6.864
$EuMn_2Sb_2$	4.570	7.660	3.926	8.341
$EuZn_2As_2$	4.211	7.181	4.219	7.088
$EuZn_2P_2$	4.087	7.010	4.069	6.907
$EuZn_2Sb_2$	4.489	7.609	4.526	7.500
Mg_3Bi_2	4.671	7.403	4.703	7.439
$SrCd_2As_2$	4.460	7.420	4.514	7.465
$SrCd_2P_2$	4.338	7.269	4.378	7.303
$SrCd_2Sb_2$	4.709	7.822	4.785	7.868
$SrMg_2As_2$	4.410	7.410	4.443	7.445
$SrMg_2Bi_2$	4.790	7.930	4.848	8.015
$SrMg_2N_2$	3.622	6.359	3.616	6.354
$SrMg_2Sb_2$	4.700	7.830	4.752	7.879
$SrMn_2As_2$	4.290	7.320	3.893	7.421
$SrMn_2P_2$	4.163	7.130	3.798	7.049

$SrMn_2Sb_2$	4.560	7.700	3.963	8.513
$SrZn_2As_2$	4.223	7.268	4.259	7.309
$SrZn_2P_2$	4.100	7.101	4.107	7.120
$SrZn_2Sb_2$	4.503	7.721	4.555	7.746
$YbCd_2Sb_2$	4.644	7.552	4.701	7.561
$YbMg_2Bi_2$	4.732	7.652	4.762	7.648
$YbMg_2Sb_2$	4.650	7.540	4.675	7.550
$YbMn_2As_2$	4.226	6.964	3.839	7.008
$YbMn_2Sb_2$	4.529	7.450	3.862	8.319
$YbZn_2As_2$	4.157	6.954	4.176	6.942
$YbZn_2P_2$	4.035	6.774	4.027	6.745
$YbZn_2Sb_2$	4.444	7.424	4.482	7.388
$SmMg_2Bi_2$	4.774	7.849	4.854	7.386
$SmMg_2Sb_2$	4.686	7.719	4.697	7.342
$CaAl_2Ge_2$	4.167	7.211	4.203	7.155
$CaAl_2Si_2$	4.137	7.131	4.157	7.102
$EuAl_2Ge_2$	4.219	7.317	4.217	7.199
$EuAl_2Si_2$	4.168	7.223	4.181	7.224
$MgAl_2Ge_2$	4.117	6.787	4.132	6.767
$MgAl_2Si_2$	4.050	6.740	4.084	6.664
$SrAl_2Ge_2$	4.225	7.448	4.260	7.483
$SrAl_2Si_2$	4.179	7.429	4.209	7.434
$YbAl_2Ge_2$	4.184	7.047	4.180	7.038
$YbAl_2Si_2$	4.140	6.950	4.147	7.040

Supplementary Table 2 The estimated m^* along Γ-K and Γ-A directions and the K value of AB_2X_2 Zintl phase compounds with p_z-dominated valence band.

	$m^*_{\Gamma\text{-K}}$ (m_e)	$m^*_{\Gamma\text{-A}}$ (m_e)	K
$YbMg_2Sb_2$	0.70	0.12	5.8
$CaMg_2Bi_2$	0.73	0.04	16.8
$CaMg_2Sb_2$	0.86	0.12	6.9
$CaMg_2As_2$	1.24	0.16	7.7
$CaMg_2N_2$	9.48	0.54	17.5
$EuMg_2Bi_2$	0.64	0.19	3.4
$EuMg_2Sb_2$	0.93	0.12	7.6
$YbMg_2Bi_2$	0.68	0.08	8.2
Mg_3Sb_2	0.90	0.11	8.0
Mg_3Bi_2	0.52	0.11	4.9
